# Adaptation of feeding behaviors on two *Brassica* species by colonizing and noncolonizing *Bemisia tabaci* (Hemiptera: Aleyrodidae) NW whiteflies

**DOI:** 10.1093/jisesa/ieae084

**Published:** 2024-09-03

**Authors:** Jaclyn S Zhou, Huaying Karen Xu, Martin Drucker, James C K Ng

**Affiliations:** Department of Microbiology and Plant Pathology, University of California, Riverside, CA, USA; Department of Statistics, University of California, Riverside, CA, USA; Virus Vector Interactions, UMR 1131 SVQV, INRAE, Université de Strasbourg, Colmar, France; Department of Microbiology and Plant Pathology, University of California, Riverside, CA, USA; Center for Infectious Disease and Vector Research, University of California, Riverside, CA, USA

**Keywords:** electrical penetration graph, insect behavior, colony-host, plant deterrence, phloem acceptance

## Abstract

*Bemisia tabaci* New World (NW) (Gennadius) (Hemiptera: Aleyrodidae), a whitefly in the *B. tabaci* species complex, is polyphagous on many plant species. Yet, it has been displaced, albeit not entirely, by other whitefly species. Potential causes could include issues with adaptation, feeding, and the colonization of new-hosts; however, insights that would help clarify these possibilities are lacking. Here, we sought to address these gaps by performing electropenetrography (EPG) recordings of NW whiteflies, designated “Napus” and “Rapa,” reared on 2 colony hosts, *Brassica napus* and *B. rapa*, respectively. Analysis of 17 probing and pathway (pw) phase-related EPG variables revealed that the whiteflies exhibited unique probing behaviors on their respective colony hosts, with some deterrence being encountered on *B. rapa*. Upon switching to *B. rapa* and *B. napus*, the probing patterns of Napus and Rapa whiteflies, respectively, adapted quickly to these new-hosts to resemble that of whiteflies feeding on their colony hosts. Results for 3 of the EPG variables suggested that *B. rapa*’s deterrence against Napus whitefly was significant prior to the phloem phase. This also suggested that adaptation by Rapa whitefly improved its pw probing on *B. rapa*. Based on analysis of 24 phloem phase-related EPG variables, Napus and Rapa whiteflies performed equally well once they entered phloem phase and exhibited comparable phloem acceptability on both the colony- and new-hosts. These findings demonstrate that NW whiteflies reared on a colony host are highly adaptable to feeding on a new host despite encountering some deterrence during the nonphloem phases in *B. rapa* plant.

## Introduction

*Bemisia tabaci* whiteflies exist as a species complex with over 40 morphologically similar species that are damaging the crops worldwide (De Barro et al. 2011, Lee et al. 2013, Hu et al. 2018, Kanakala and Ghanim 2019), and are polyphagous on over 1,000 plant species (Li et al. 2021). Adult whiteflies and all nymphal instars use their piercing-sucking mouthparts to ingest phloem sap nutrients located in the sieve elements of plants on which they feed. Concomitantly, honeydew secretions produced and deposited on the plant surface by their feeding activities attract and promote the growth of sooty mold. Known by the moniker “stealthy phloem feeders,” whiteflies avoid causing severe mechanical damages to plants while feeding with their flexible stylet bundles. This is achieved by covertly maneuvering their stylets intercellularly through plant tissues and rarely puncturing cells until they are close to locating the phloem (Janssen et al. 1989, Walker and Perring 1994, Jiang et al. 1999, Johnson and Walker 2003). In addition, they secrete gelling and watery saliva that function in tempering plant defense responses (Xu et al. 2019, Li et al. 2023). The interplay of both mechanical and chemical actions between the whitefly salivary and plant defense components may be similar to that between other phloem feeders, e.g., aphids and plants (Walling 2000, Wang et al. 2017, Naalden et al. 2021). With such sophistication devoted to avoiding and mitigating host defenses, it is no surprise that there is much to be learned about the feeding behaviors of whiteflies, including those in the *B. tabaci* species complex. In this respect, the use of electropenetrography (EPG; a.k.a. electrical penetration graph monitor) (Supplementary Fig. S1)—a noninvasive technique based on the principles of electrical circuitry—to track the movement (a.k.a. probing) of the stylet bundles as well as feeding activities is instrumental for investigating the unique interactions between the whitefly and the plant (Mclean and Kinsey 1964, Tjallingii 1978, Walker 2000, Backus et al. 2016). In an EPG system, when contact is made between the insect and plant, current flows through the circuit and waveforms that correspond to different feeding behaviors are generated in real time.

Feeding behaviors are fundamental to the processes underlying the transmission of insect vectored plant viruses (Dader et al. 2017, Zhou et al. 2018, Maluta et al. 2019). Accordingly, the unique feeding behaviors of whiteflies in the *B. tabaci* complex have contributed to their success as vectors of many economically important plant viruses (Fiallo-Olivé et al. 2020). While *B. tabaci* Middle East-Asia Minor 1 (MEAM1; formerly *B. tabaci* biotype B and *B. argentifolii*) and Mediterranean (MED; formerly *B. tabaci* biotype Q) vectors have spread globally, the less well studied *B. tabaci* New World (NW; formerly *B. tabaci* biotype A) vector still exists in the Americas and has not been completely displaced (Barbosa Lda et al. 2014, De Marchi et al. 2017, Can-Vargas et al. 2020). Most notably, studies using NW vectors, which transmit specific viruses in the genus *Crinivirus* (family *Closteroviridae*) (Chen et al. 2012, De Marchi et al. 2017, Fiallo-Olivé and Navas-Castillo 2023), have provided important insights into the molecular interactions that mediate crinivirus transmission. For example, the transmission of lettuce infectious yellowing virus (LIYV) is determined by a capsid protein (the LIYV minor coat protein [CPm]) mediated virion retention mechanism in the anterior foregut of the NW vector (Chen et al. 2011, Ng 2013). Two CPm domains involved in mediating virion binding to the NW vector’s foregut have also been identified (Ng et al. 2021). Knowledge gained from studying the feeding behaviors of the NW vector would contribute immensely to understanding the transmission mechanisms of criniviruses and other foregut-borne viruses (Ng and Zhou 2015, Zhou et al. 2018).

Whiteflies are adept to overcoming specific physical and chemical barriers and, being polyphagous, have varying degrees of success persisting on different plants (Van Lenteren and Noldus 1990). For example, substantial studies had established a list of suitable plants (ranked from the most to the least favorable) for *Trialeurodes vaporariorum* (the greenhouse whitefly) according to the following criteria: longevity, mortality, fecundity, developmental time, and oviposition (Van Lenteren and Noldus 1990). By contrast, fewer studies have examined the feeding (probing) behaviors of the greenhouse whitefly; those that did reported that it generally fed better on cucumber plant than on tomato and sweet pepper plants, and this was consistent with the findings of the plant suitability ranking studies (Lei et al. 1998, 2001, Liu et al. 2013). A range of suitable plants have also been observed for different species of *B. tabaci* (Burban et al. 1992, Legg 1996). While the SSA1 to 5 species of *B. tabaci* are colonizers of cassava plants, they may also be able to colonize other plants. In contrast, other species of the *B. tabaci* complex—MEAM1, MED, and NW—have never been found on cassava plants (Brown et al. 1995, Legg 1996, Carabalí et al. 2010, De Barro et al. 2011). Colonization refers to the ability of an insect to complete all its developmental stages on a plant, leading to the establishment of a viable population. An EPG study of the cassava-colonizing *B. tabaci* SSA1-SG3 showed that it also feeds well on sweet potato and cotton plants, but less effectively on tomato plants; whereas *B. tabaci* MED feeds poorly on cassava plants (Milenovic et al. 2019). In other studies, MEAM1 feeds better on cabbage and cucumber plants than MED, but the opposite is observed on tomato, cotton and poinsettia plants. Conversely, both MEAM1 and MED feed poorly on pepper plants (Liu et al. 2012, Jiao et al. 2014).

The question of whether or not a whitefly species reared on a particular host plant could perform (feed/probe) better on that plant than another whitefly (of the same species) that has been reared on a different host plant has rarely been examined. Based on available information, *B. tabaci* tends to prefer the host plant that it colonizes (Brown et al. 1995), although it is generally accepted that *B. tabaci* is polyphagous rather than monophagous (De Barro et al. 2011). Also, unlike for other whitefly species and species in the *B. tabaci* complex (Janssen et al. 1989, Lei et al. 1997, Jiang et al. 1999, Jiang and Walker 2001, Liu et al. 2012, Jiao et al. 2014, Milenovic et al. 2019), the plant-feeding behaviors of NW have never been examined. In this study, we reared NW on 2 *Brassica* plant species, *B. napus* and *B. rapa*, generating 2 colonies of whiteflies by allowing them to adapt to each plant species for 6 and 4 years, respectively. From here on, whiteflies from the *B. napus* colony will be referred to as Napus (N) whiteflies and those from the *B. rapa* colony will be referred to as Rapa (R) whiteflies. Plants on which whiteflies were reared will be referred to as colony-hosts or colony-host plants. Using the EPG monitor, we studied the feeding behaviors of Napus and Rapa whiteflies on their respective colony-hosts *B. napus* and *B. rapa*. We also examined the feeding behaviors of Napus and Rapa whiteflies on *B. rapa* and *B. napus*, respectively, to determine their ability to adapt to an unfamiliar, new-host plant. Our studies showed that Napus and Rapa whiteflies exhibited a unique probing pattern on their respective colony-hosts. Even after years of being reared on a particular colony-host, Napus and Rapa whiteflies could quickly adapt to an unfamiliar, new-host*. B. rapa* plant showed some deterrence towards Rapa whiteflies during probing and before the phloem phase, and the level of deterrence was amplified for Napus whiteflies. In terms of phloem acceptability, Napus and Rapa whiteflies performed equally well on both plants.

## Materials and Methods

### Whitefly Colonies and Plants

*Bemisia tabaci* NW whiteflies were originally obtained from a field collection (Perring et al. 1993), and subsequently maintained on lima bean (*Phaseolus limensis*) plants (Tian et al. 1999). NW whiteflies from the culture maintained on lima bean plants were used to establish the Napus and Rapa whitefly colonies on *B. napus* or *B. rapa* plants, respectively. The Napus and Rapa whitefly colonies were continuously maintained for 6 and 4 years, respectively, prior to use in EPG experiments. The whitefly colonies were kept in separate Bugdorm 2120-F insect rearing tents and separate rooms (in the UC Riverside Insect and Quarantine facility) maintained at a constant temperature of 85°F, 30% humidity, and a photoperiod of 16 h light/8 h dark. Adult whiteflies used for EPG experiments were collected from a plant 13 to 17 days after it had been added to a colony. *B. napus* plants (seeds were originally a gift from Julia Kehr, University of Hamburg) and *B. rapa* (Hakurei F1) plants (seeds purchased from Johnny’s Seeds, Winslow, Maine, USA) used for maintaining whitefly colonies were grown in UC mix 3 soil (https://agops.ucr.edu/soil-mixing) under the same conditions used for rearing the whiteflies. Plants used for EPG experiments were grown in UC mix 3 soil under greenhouse conditions and used 2 wk after germination.

### Whitefly Wiring and EPG Recording

EPG analysis of a female whitefly was performed using a 2.5-µm diameter platinum wire (Wollaston process wire, Sigmund Cohn Corp., Mt. Vernon, NY, USA) (Supplementary Fig. S1). Prior to use, the platinum wire was prepared in the following steps: (i) one end of a 2-cm silver encased platinum wire was attached to the head of a 3-mm diameter nail using silver glue (Electrodag 503, Ladd Research Industries, Williston, Vermont); (ii) silver glue was brushed (less than 1 mm) onto the other end of the wire at the tip; (iii) half the length (i.e., 1 cm) of the silver coated platinum wire was dipped into a petri dish containing 50% nitric acid to dissolve the silver casing; (iv) the exposed platinum wire was dipped in water to remove residual nitric acid. After the wire preparation, a tube containing a mix of male and female whiteflies harvested from a Napus or Rapa whitefly colony was chilled in a −20 °C freezer for 35 s to immobilize the whiteflies. The chilled whiteflies were kept immobilized by transferring them onto a petri dish placed on top of a cold plate. A 1 × 1 cm plastic sheet was placed inside the petri dish near the immobilized whiteflies and a drop of silver glue was placed onto the plastic sheet. With the petri dish under the view of the stereomicroscope (Nikon SMZ445T), the exposed end of the platinum wire was first dipped into the silver glue on the plastic sheet and immediately placed on top of the thoracic dorsum of a female whitefly (Supplementary Fig. S1).

EPG recordings were carried out using an 8-channel Giga-8D DC-EPG monitor with a 1 Giga-ohm input resistance (EPG systems, Wageningen, The Netherlands) (Supplementary Fig. S1). A wired female whitefly was allowed to feed for at least 4 h on the abaxial side of a leaf on a potted 2 wk old *B. napus* or *B. rapa* plant. The substrate voltage was applied into the soil of the plant pot and the substrate voltage was adjusted to fit the + 6- to −6-volt vertical axis on the Stylet + daq acquisition software (EPG systems, Wageningen, The Netherlands). The default standard sample frequency of 100 Hz was selected. Twenty EPG recordings each were completed for Napus whiteflies placed onto *B. napus* plants and for Rapa whiteflies placed onto *B. rapa* plants. Twenty-one EPG recordings each were completed for Napus whiteflies placed onto *B. rapa* plants and for Rapa whiteflies placed onto *B. napus* plants. The order of the treatments on the 8 channels was randomized in every experiment. All experiments were performed inside a Faraday cage (Supplementary Fig. S1). Lights were provided above the Faraday cage and temperature was maintained at 24 °C.

### Analysis and Statistics

EPG recordings were annotated using EPG Stylet + analysis software (https://www.epgsystems.eu/) for the following feeding behavior waveforms: nonprobing, pathway (pw), xylem ingestion, potential drops (pd), phloem salivation (E1), and ingestion (E2). Waveforms F, suggesting derailed stylet mechanics (typically observed in the pw phase), were absorbed into the pw phases of the respective recordings. For EPG recordings in which phloem ingestion (E2) was artificially terminated at the 4 h mark of a feeding event, annotation continued until the E2 phases were completed or for up to a maximum of 6 h, depending on whichever came first (Ebert et al. 2018). The annotated files were imported into an excel analysis software (Sarria et al. 2009) and 41 variables relevant to whitefly behaviors were selected for statistical analysis using Graphpad Prism 10 (GraphPad Software, Boston, MA, USA). Each feeding behavior variable was compared using a one-way ANOVA followed by Tukey’s Multiple Comparisons Test (MCT). Feeding variables that did not meet normality and homogeneity of variances were log or square root transformed, and reanalyzed. If the transformed data did not meet the assumptions of normality and homogeneity, data were reanalyzed using the nonparametric Kruskal–Wallis test and Dunn’s MCT. Fisher’s exact test was used to compare the differences in the number of whiteflies that reached phloem phase among the treatments.

## Results

### EPG Waveforms

**Fig. 1.**
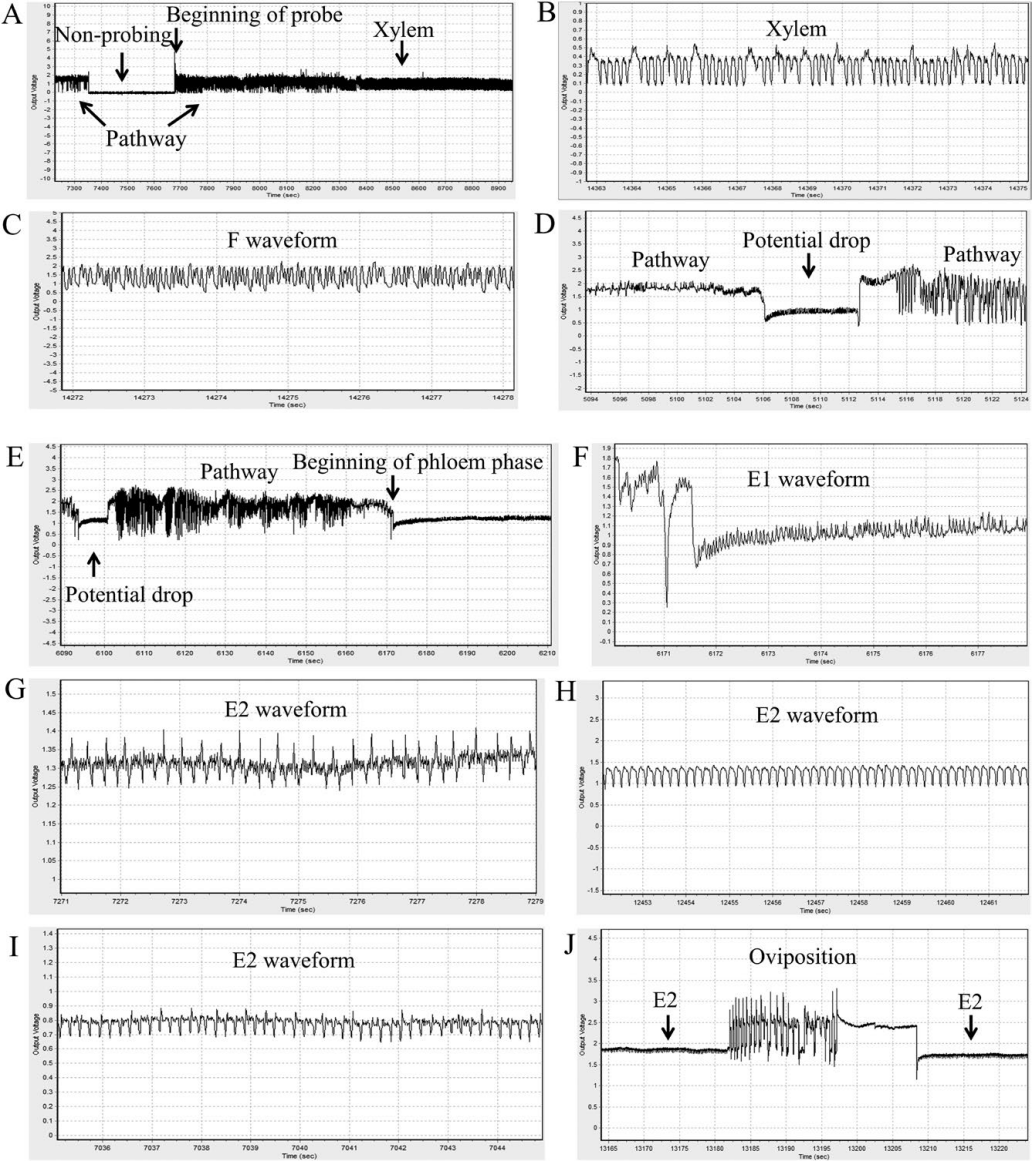
Representative waveforms from electropenetrography (EPG) recordings of whiteflies (*Bemisia tabaci* New World [NW]) feeding on *Brassica napus* and *B. rapa* plants. The vertical axis is the output voltage, with zero volts (nonprobing) being the reference point. The horizontal axis is time in seconds, with each division corresponding to 100 s (A), 1 s (B, C, F, G, H, I), 2 s (D), 5 s (J), and 10 s (E). A) The beginning of a probe with pathway (pw) phase followed by transition into xylem phase. B) Xylem waveform. C) F waveform (derailed stylets). D) Potential drop (pd; intracellular punctures) indicated by an arrow. E) Potential drops and pw phase before beginning of phloem phase (indicated by arrows). F) E1 (salivation) waveform (detailed view of E). G–I) Three types of E2 (ingestion) waveforms. J) Oviposition during phloem ingestion.

This is the first reported EPG study of *B. tabaci* NW on plants. Regardless of the combination of NW whitefly colony (Napus or Rapa) and plant (*B. napus* or *B. rapa*) used in the EPG recordings, the waveform patterns observed in the EPG studies of other whiteflies were also seen in our study (Fig. 1) (Janssen et al. 1989, Jiang et al. 1999, Johnson and Walker 2003, Liu et al. 2012, Zhou 2014, Milenovic et al. 2019). Similar to other whitefly EPG studies, waveforms in the pw phase (which corresponds to the intercellular movement of the stylet bundle) had many variations for which the exact behavioral correlations remain unknown. Extracellular xylem ingestion and F waveforms (corresponding to derailed stylets) were also observed in the recordings (Fig. 1B and C). Xylem waveforms were always preceded by pw phase waveforms and were identified by the absence of a voltage drop transitioning from pw to xylem (Fig. 1A). The xylem waveforms had a steady amplitude and a frequency of 5–7 Hz/s. This contrasted with the F waveforms, which were at a higher frequency, ranging from 12 to 18 Hz/s. Whiteflies infrequently made intracellular cell punctures early in pw phases and these were observed as pd waveforms in the recordings (Fig. 1D). More often, pd were observed only when the whitefly’s stylet bundle had reached deep into plant tissues near the phloem (Fig. 1E). Upon entering the phloem phase (E), as was indicated the by a drop in voltage, whiteflies salivated (seen as an E1 waveform) into sieve element (Fig. 1F) before initiating sap ingestion (seen as E2 waveforms). Variations of phloem ingestion, seen as 3 different types of E2 waveforms, were observed and this was consistent with previous studies as well (Fig. 1G–I). Oviposition was observed in pw phases (not shown) as well as during phloem ingestion when a Napus or Rapa whitefly was placed on either of the 2 *Brassica* species (Fig. 1J).

### Distinct Probing Behaviors on a Colony-Host

**Fig. 2.**
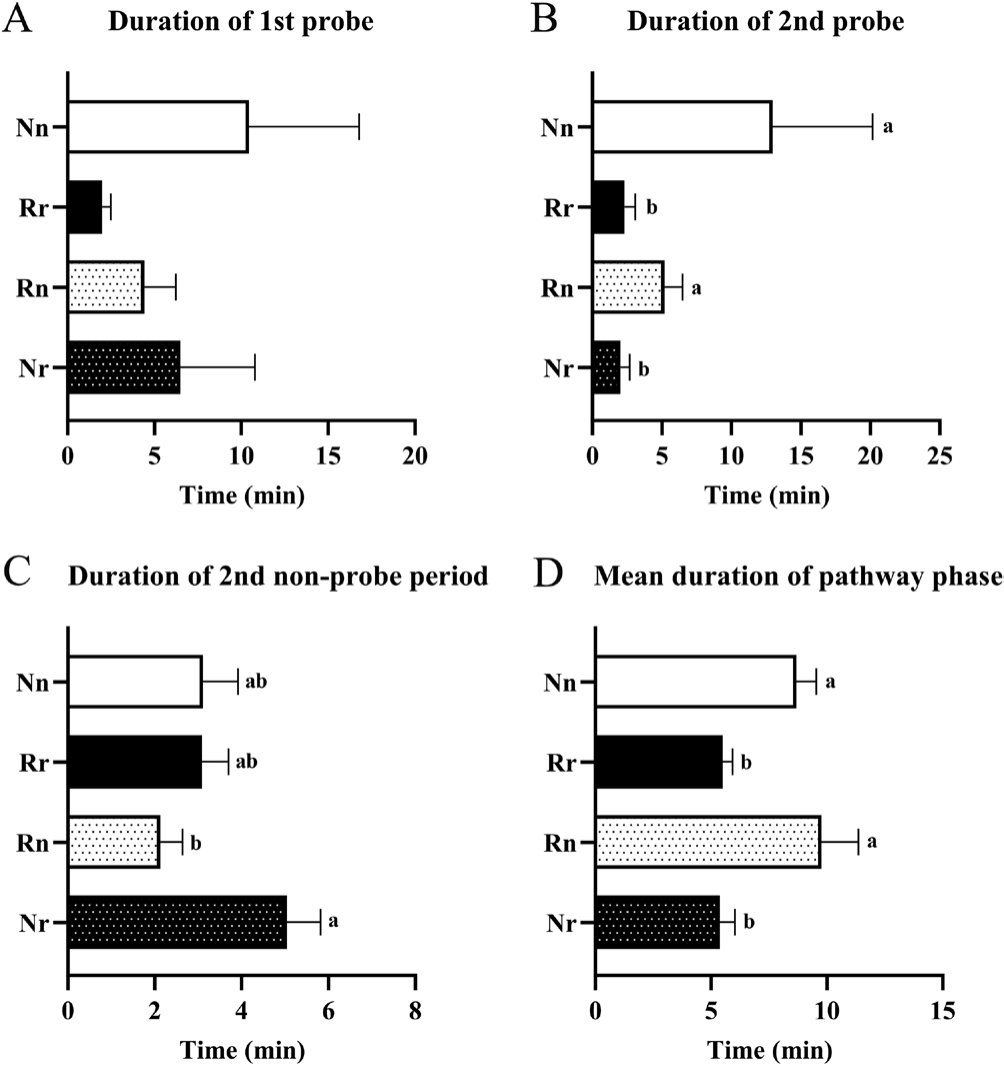
Analysis of 4 EPG variables, 3 of which being associated with early probes and the 4th being the mean duration of pathway phase of Napus and Rapa whiteflies feeding on *Brassica napus* and *B. rapa* plants. Data (time in minutes) are mean ± standard deviation for the following treatments: (i) Nn; Napus whiteflies on *B. napus* plants (white bars) (*n* = 20), (ii) Rr; Rapa whiteflies on *B. rapa* plants (black bars) (*n* = 20), (iii) Rn; Rapa whiteflies on *B. napus* plants (white-spotted bars) (*n* = 21), and (iv) Nr; Napus whiteflies on *B. rapa* plants (black-spotted bars) (*n* = 21). Significant difference among the treatments is indicated by different letters. *P* values for each EPG variable analyzed are from A) Nonparametric Kruskal-Wallis Test (*P* = 0.12), B) one-way ANOVA of log10 transformed data [*F*_(3, 78)_ = 6.0; *P *= 0.001] and Tukey’s MCT, C) one-way ANOVA of log10 transformed data [*F*_(3, 78)_ = 3.5; *P *= 0.02] and Tukey’s MCT, D) Nonparametric Kruskal–Wallis test & Dunn’s MCT (*P *< 0.0001).

**Fig. 3.**
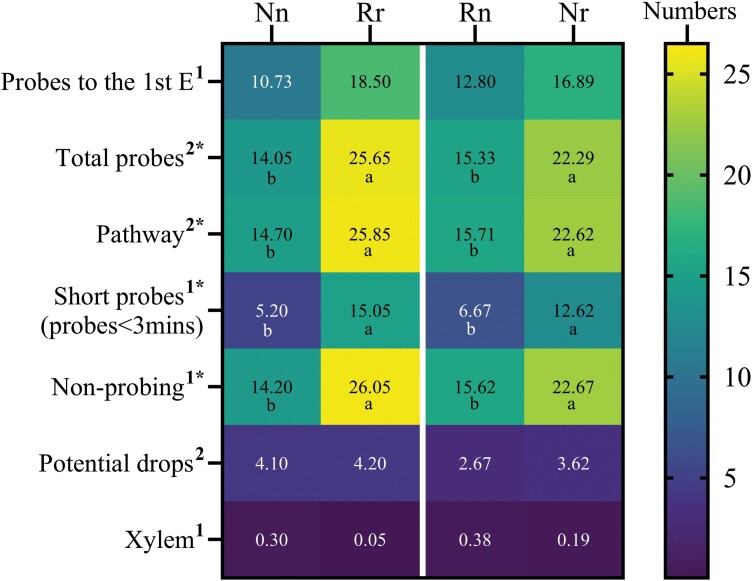
Heat map analysis of 7 EPG variables associated with the pathway (nonphloem) phases and probing behaviors of Napus and Rapa whiteflies on *Brassica napus* and *B. rapa* plants. Each row in the heat map is an EPG variable, and each column represents the EPG recording of one of the following treatments: (i) Nn; Napus whiteflies on *B. napus* plants, (ii) Rr; Rapa whiteflies on *B. rapa* plants, (iii) Rn; Rapa whiteflies on *B. napus* plants, and (iv) Nr; Napus whiteflies on *B. rapa* plants. The heat map was created using data (numbers) obtained for each variable and presented as the mean ± standard deviation (*n* = 20 [treatments 1 and 2]; *n* = 21 [treatments 3 and 4]). The only exception is the first variable (Probes to the 1st E), where *n* = 11, 8, 10, and 9 for treatments 1, 2, 3, and 4, respectively. Means are indicated within each colored square of the heat map. Nonparametric Kruskal–Wallis test and Dunn’s MCT, or Parametric one-way ANOVA on log10 transformed data and Tukey MCT, indicated next to each variable by the superscripts 1 and 2, respectively, were used to obtain the *P* values. Significant *P* values (< 0.0001) are indicated by an asterisk. Significant differences among the treatments are indicated by different letters.

**Fig. 4.**
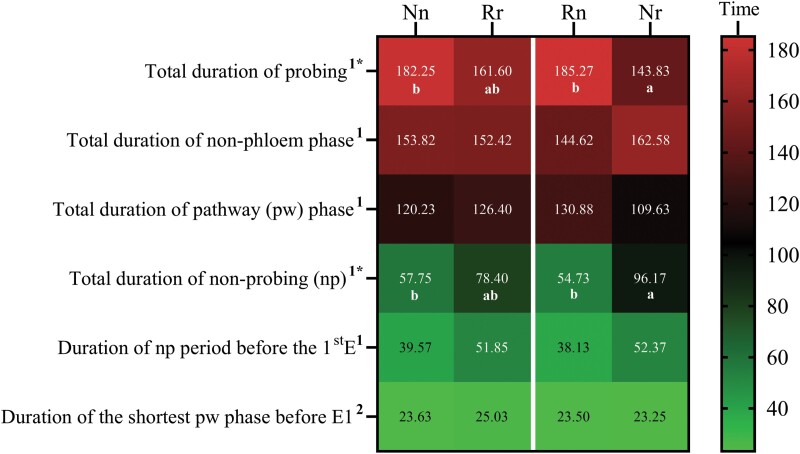
Heat map analysis of 6 EPG variables associated with the duration of pathway (pw) phases and probing behaviors of Napus and Rapa whiteflies on *Brassica napus* and *B. rapa* plants. Each row in the heat map is an EPG variable, and each column represents the EPG recording of one of the following treatments: (i) Nn; Napus whiteflies on *B. napus* plants, (ii) Rr; Rapa whiteflies on *B. rapa* plants, (iii) Rn; Rapa whiteflies on *B. napus* plants, and (iv) Nr; Napus whiteflies on *B. rapa* plants. The heat map was created using data (time in minutes) obtained for each variable and presented as the mean ± standard deviation (*n* = 20 [treatments 1 and 2]; *n* = 21 [treatments 3 and 4] for the following variables: total duration of probing, total duration of pw phase, and total duration of np). For the other remaining variables, *n* = 11, 8, 10, and 9, respectively, for treatments 1, 2, 3, and 4. Means are indicated within each colored square of the heat map. Parametric one-way ANOVA of data and Tukey’s MCT, or nonparametric Kruskal–Wallis test and Dunn’s MCT, indicated next to each variable by the superscripts 1 and 2, respectively, were used to obtain the *P* values. Significant *P* values (< 0.001) are indicated by an asterisk. Significant differences among the treatments are indicated by different letters.

EPG recordings and analysis of whitefly feedings were performed using 4 different treatments—(i) Napus whiteflies on *B. napus* plants (Nn), (ii) Rapa whiteflies on *B. rapa* plants (Rr), (iii) Rapa whiteflies on *B. napus* plants (Rn), and (iv) Napus whiteflies on *B. rapa* plants (Nr). For simplicity, whiteflies subjected to these feeding treatments were given the acronyms shown in the parentheses, where the upper-case letter represents the (N)apus or (R)apa whitefly, and the lower-case letter represents the *Brassica* species, *napus* (n) or *rapa* (r), on which EPG recordings were made. Seventeen variables relevant to probing behaviors—e.g., duration of the first probe, duration of the second probe (Fig. 2); probes to the first E, total probes (Fig. 3); total duration of probing, total duration of nonphloem phase (Fig. 4)—were analyzed for treatments 1 and 2 to compare the probing behaviors exhibited by whiteflies while they were feeding on their respective colony-hosts (i.e., Nn and Rr). Nn and Rr each exhibited a unique feeding behavioral pattern. Nn showed a longer duration of the first probe and a significantly longer duration of the second probe (*P *= 0.039) than did Rr (Fig. 2A and B). Because whiteflies used for EPG recordings were obtained from colonies adapted to these plants, the significant difference was unlikely related to the need for acclimation. The duration of the second nonprobing period is a measure of host deterrence (Van Helden and Tjallingii 2000); a short duration suggests less deterrence while a long duration suggests more deterrence. Nn and Rr did not differ significantly (*P* = 0.98) in their second nonprobing periods (Fig. 2C), suggesting that they were not deterred from continuing to feed on the plants. Consistent with the early probes data (Fig. 2A–C), Nn exhibited a significantly (*P* = 0.007) longer mean duration of pw phases than did Rr (Fig. 2D). In a composite of 7 EPG variables analyzed for pw phase and probing behaviors, whiteflies showed a distinct feeding pattern when navigating through the tissue layers of their colony-hosts (Fig. 3). The feeding behaviors of Nn, compared to that of Rr, were characterized by significantly fewer of the following: short probes (< 3 min) (*P* < 0.0001), pw (*P* = 0.001), nonprobing (*P* = 0.0006), and total probes (*P* = 0.0005) (Fig. 3). On the same trend, it took Nn fewer probes than Rr (10.73 vs 18.5) to reach the first phloem phase (E) even though the difference was not significant (*P* = 0.67).

These results, along with a significantly longer (*P* = 0.007) mean pw phase of Nn relative to that of Rr (Fig. 2D), would generally be seen as a higher level of host acceptance by Nn than by Rr. However, data analysis of additional variables suggested that Nn and Rr displayed similar host acceptance in due course (Figs. 3 and 4). First, the number of both pd (corresponding to cell punctures) (*P *= 0.97) and entries into xylem phase (*P* = 0.64) were similar between Nn and Rr (Fig. 3). Second, data on the total duration of both pw phase (*P *= 0.97) and nonprobing (*P* = 0.27) of the respective colony-hosts were not significantly different between Nn and Rr (Fig. 4). Furthermore, the total duration of nonphloem phase (specific to recordings of whiteflies that reached phloem phase), which accounted for the total time spent in pw and nonprobing, was nearly identical between Nn (153.82 min) and Rr (152.42 min) (*P* > 0.99) (Fig. 4). The conclusion that both Nn and Rr exhibited similar levels of host acceptability is further supported by data from variables that examined the duration of nonprobing period before the first phloem phase (*P* = 0.84) and duration of the shortest pw phase before E1 salivation (*P* > 0.99) (Fig. 4), wherein no significant difference was observed. Further, although the total probing time, which included time spent in the pw and phloem phases (excluding nonprobing time), was longer for Nn than for Rr, the difference was not significant (*P *= 0.27) (Fig. 4).

### Whitefly on a New-Host Takes on the Feeding Patterns of Whitefly on a Colony-Host

To compare the feeding behaviors of whiteflies on new-hosts, the same 17 EPG variables analyzed for Nn and Rr (treatments 1 and 2; whiteflies on colony hosts) in the preceding section were examined for Rn and Nr (treatments 3 and 4; whiteflies on new-hosts ) (Figs. 2–4). During the early probes, both Napus and Rapa whiteflies were able to distinguish the new-hosts and quickly adjusted their probing behaviors accordingly (Fig. 2). Several key observations led to this conclusion. First, although the duration of the first probe on the colony hosts was longer for Nn than Rr, this pattern was not observed on the new-hosts for Rn and Nr (Fig. 2A). Second, however, by the second probe, Nr quickly adapted to *B. rapa* plants, and the duration of this probe reduced significantly (*P *= 0.037), resembling that of Rr (Fig. 2B). The duration of the second probe of Rn also resembled that of Nn in being significantly (*P *= 0.009) longer than that of Nr (Fig. 2B). Third, the duration of the second nonprobe period was significantly longer (*P* = 0.01) for Nr than Rn (Fig. 2C). This could be due to whiteflies either encountering more deterrence from, or undergoing an initial brief period adapting to, the new-hosts. Finally, the mean durations of the pw phase of Rn and Nr mirrored that of Nn and Rr, i.e., being longer on *B. napus* plants than on *B. rapa* plants (*P* < 0.0001) regardless of whether the whiteflies were on the colony-hosts or new-hosts (Fig. 2D).

Regardless of the whiteflies used, the plant on which EPG was performed dictated the pattern of probing behaviors. This was evidenced by data from the heat map analysis of 5 EPG variables associated with pw phases and probing behaviors (Fig. 3). First, the probing patterns of Rn were comparable to that of Nn (Fig. 3), while Nr took on similar probing patterns as Rr (Fig. 3). Second, the number of short probes (< 3 min) (*P* = 0.03), pw probes (*P* = 0.01), nonprobing (*P* = 0.029), and total probes (*P* = 0.01) were significantly higher on *B. rapa* plants than on *B. napus* plants even when whiteflies were unfamiliar to these new-hosts. Similar to whitefly feeding on colony-hosts, whitefly feeding on new-hosts was not associated with a significant number of pd (*P *= 0.80) and xylem entry phases (*P* > 0.99), suggesting that these variables were neutral in terms of dictating the feeding patterns of the whiteflies. Data from the heat map analysis of 4 EPG variables relevant to pw phases and probing behaviors (Fig. 4) were consistent with those presented in Fig. 3 in supporting the observation that whiteflies on new-hosts took on the feeding patterns of those on colony hosts (Fig. 4; Nn and Rn vs Rr and Nr).

Interestingly, a significant difference was observed between data from 2 of the variables—the total duration of probing and nonprobing—when whiteflies were not adapted to the new-hosts (Fig. 4; Rn vs Nr). Specifically, the total probing duration, which takes into account time spent in the pw and phloem phases but excludes the nonprobing time, was significantly longer (*P* = 0.002) for Rn than Nr. The reverse was seen in the total nonprobing duration, where it was significantly longer (*P* = 0.002) for Nr than Rn. The implications of these results will be explained in the Discussion section.

### Whiteflies Exhibit Comparable Phloem Acceptability on Colony- and New-Hosts

We next analyzed the data from treatments 1 to 4 (Nn, Rr, Rn, and Nr) for EPG variables that can be used to evaluate a whitefly’s ability to achieve phloem phase (where salivation and sap ingestion take place) and to assess the phloem acceptability of colony- and new-hosts (Tables 1 and 2). Regardless of the whiteflies (Napus or Rapa whiteflies) and the plants (colony-host or new-host) on which EPG recordings were done, the variables related to the durations to achieve the following events (shown in Table 1): the first phloem phase (E) (variables 1–3), the first ingestion phase (E2) (variables 4–6), and sustained phloem phase (ingestion longer than 10 min) (variables 7–9), were all not significantly different (*P* values in Table 1). In addition, the time from the beginning of the first probe to the first pd (variable 10), which usually indicates that a whitefly is close to reaching phloem phase (thus a strong indicator of success in reaching phloem phase), was not significantly different (*F*_(3, 48)_ = 0.1; *P* = 0.93) among all 4 treatments. We also compared the number of whiteflies that reached phloem phase in all 4 treatments and the results were not significantly different (11/20 for Nn, 8/20 for Rr, 10/21 for Rn, and 9/21 for Nr) (Fisher’s exact test, *P* = 0.80).

**Table 1.** Analysis of EPG variables for assessing a whitefly’s ability to reach phloem phase

Variables Time taken by whitefly from:	Nn Mean ± *SD* (*n*)	Rr Mean ± *SD* (*n*)	Rn Mean ± *SD* (*n*)	Nr Mean ± *SD* (*n*)	*P* values^d^
1. being placed on plant to reaching 1st E^a^	169.7 ± 81.6 (20)	195.0 ± 69.8 (20)	182.3 ± 74.7 (21)	195.0 ± 63.9 (21)	0.72^**1**^
2. making 1st probe to reaching 1st E	169.0 ± 81.5 (20)	191.7 ± 68.6 (20)	181.5 ± 74.4 (21)	193.7 ± 63.7 (21)	0.82^**1**^
3. making probe that reached 1st E to achieving 1st E	26.4 ± 12.3 (11)	25.0 ± 14.2 (8)	23.5 ± 11.2 (10)	23.3 ± 9.1 (9)	0.94^**2**^*F*_(3, 34)_ = 0.1
4. being placed on plant to reaching 1st E2^b^	170.0 ± 81.4 (20)	195.2 ± 69.6 (20)	182.5 ± 74.5 (21)	195.8 ± 63.3 (21)	0.71^**1**^
5. making 1st probe to reaching 1st E2	169.3 ± 81.3 (20)	191.9 ± 68.4 (20)	181.7 ± 74.2 (21)	194.5 ± 63.1 (21)	0.81^**1**^
6. making probe that reached 1st E2 to achieving 1st E2	26.9 ± 12.4 (11)	25.5 ± 14.3 (8)	23.9 ± 11.2 (10)	25.2 ± 9.4 (9)	0.95^**3**^*F*_(3, 34)_ = 0.1
7. being placed on plant to reaching 1st sustained E2	170.0 ± 81.4 (20)	195.2 ± 69.6 (20)	182.5 ± 74.5 (21)	195.8 ± 63.3 (21)	0.71^**1**^
8. making 1st probe to reaching 1st sustained E2	169.3 ± 81.3 (20)	191.9 ± 68.4 (20)	181.7 ± 74.2 (21)	194.5 ± 63.1 (21)	0.81^**1**^
9. making probe that reached 1st sustained E2 to achieving 1st sustained E2	31.3 ± 17.1 (11)	25.5 ± 14.3 (8)	23.9 ± 11.2 (10)	25.2 ± 9.4 (9)	0.61^**3**^*F*_(3, 34)_ = 0.6
10. making 1st probe to reaching first pd^c^	116.4 ± 71.8 (13)	96.2 ± 54.9 (12)	98.2 ± 55.4 (13)	116.9 ± 63.5 (14)	0.93^**2**^*F*_(3, 48)_ = 0.1

The EPG variables are shown in the first column on the left. The treatments: (i) Nn; Napus whiteflies on *Brassica napus* plants, (ii) Rr; Rapa whiteflies on *B. rapa* plants, (iii) Rn; Rapa whiteflies on *B. napus* plants, and (iv) Nr; Napus whiteflies on *B. rapa* plants are shown in the subsequent columns. Table entries (time in minutes) are mean ± standard deviation (*SD*) with *n* (number of recordings) shown in parentheses.

^a,b,c^Abbreviations: phloem phase (E), ingestion (E2), and potential drop (pd). Sustained E2 is defined as ingestion longer than 10 min.

^d^ *P* values were obtained from the following statistical analysis (indicated by the superscript numbers next to the *P* values): (i) Nonparametric Kruskal–Wallis Test (ii) Parametric one-way ANOVA of log10 transformed data or (iii) Parametric one-way ANOVA of raw data. The *F*-values and degrees of freedom entered below some of the *P* values are generated from a one-way ANOVA analysis.

**Table 2.** Analysis of EPG variables for assessing whitefly’s phloem acceptability

Variables	Nn Mean ± *SD* (*n*)	Rr Mean ± *SD* (*n*)	Rn Mean ± *SD* (*n*)	Nr Mean ± *SD* (*n*)	*P* values^d^
1. Number of E1^a^	0.9 ± 1.1 (20)	0.6 ± 0.8 (20)	0.6 ± 0.7 (21)	0.5 ± 0.6 (21)	0.56^**1**^
2. Duration of the E1 immediately before the 1st E2^b^	31.4 ± 15.2 (11)	28.2 ± 12.9 (8)	24.4 ± 9.5 (10)	22.6 ± 9.5 (9)	0.65^**1**^
3. Duration of the E1 immediately before the 1st sustained E2	26.9 ± 12.3 (11)	28.2 ± 12.9 (8)	24.4 ± 9.5 (10)	22.6 ± 9.5 (9)	0.91^**1**^
4. Total duration of E1 (that has an E2 immediately after it)	52.5 ± 41.1 (11)	39.6 ± 27.1 (8)	27.3 ± 10.9 (10)	22.6 ± 9.5 (9)	0.23^**1**^
5. Total duration of E1	52.5 ± 41.1 (11)	39.6 ± 27.1 (8)	27.3 ± 10.9 (10)	23.8 ± 10.2 (9)	0.34^**1**^
6. Mean duration of E1	27.9 ± 10.8 (11)	28.7 ± 14.6 (8)	24.0 ± 9.9 (10)	21.9 ± 9.6 (9)	0.53^**2**^*F*_(3, 34)_ = 0.7
7. Number of E2	0.9 ± 1.1 (20)	0.6 ± 0.8 (20)	0.6 ± 0.7 (21)	0.4 ± 0.5 (21)	0.51^**1**^
8. Number of sustained E2	0.7 ± 0.7 (20)	0.6 ± 0.8 (20)	0.5 ± 0.6 (21)	0.4 ± 0.5 (21)	0.79^**1**^
9. Total duration of E2	150.7 ± 97.4 (11)	191.9 ± 65.4 (8)	172.8 ± 98.3 (10)	153.3 ± 58.2 (9)	0.70^**1**^
10. Mean duration of E2	114.8 ± 104.8 (11)	155.5 ± 80.8 (8)	163.7 ± 104.5 (10)	153.3 ± 58.2 (9)	0.42^**1**^
11. Duration of the longest E2	143.6 ± 99.6 (11)	176.6 ± 69.1 (8)	169.1 ± 100.2 (10)	153.3 ± 58.2 (9)	0.83^**1**^
12. % E2 > 10 min	80.3 ± 28.7 (11)	100.0 ± 0.0 (8)	95.0 ± 15.8 (10)	100.0 ± 0.0 (9)	0.05^**1**^
13. Duration of 1st E^c^	106.4 ± 113.3 (11)	134.8 ± 102.0 (8)	160.1 ± 108.7 (10)	153.7 ± 58.1 (9)	0.41^**1**^
14. Total duration of E	151.6 ± 97.4 (11)	192.6 ± 65.5 (8)	173.3 ± 98.3 (10)	153.7 ± 58.1 (9)	0.71^**1**^

The EPG variables are shown in the first column on the left. The treatments: (i) Nn; Napus whiteflies on *B. napus* plants, (ii) Rr; Rapa whiteflies on *B. rapa* plants, (iii) Rn; Rapa whiteflies on *B. napus* plants, and (iv) Nr; Napus whiteflies on *B. rapa* plants are shown in the subsequent 4 columns. Table entries (numbers, duration in minutes, and percentages) are mean ± standard deviation (*SD*) with *n* (number of recordings) shown in parentheses.

^a, b, c^Abbreviations: salivation (E1), ingestion (E2), and phloem phase (E). Sustained E2 is defined as ingestion longer than 10 min.

^d^ *P* values were obtained from the following statistical analysis (indicated by the superscript numbers next to the *P* values): (i) Nonparametric Kruskal–Wallis test (ii) Parametric one-way ANOVA of raw data; with the *F*-value and degrees of freedom as shown below the *P* value.

Within phloem phase, 14 variables were analyzed to further assess phloem acceptability, with 6 of these variables being relevant to salivation (E1) and 6 others being relevant to phloem ingestion (E2) (Table 2, variables 1–12). In all 6 E1 related variables shown in Table 2: (i) number of E1, (ii) duration of E1 immediately before the first E2, (iii) duration of E1 immediately before the first sustained E2, (iv) total duration of E1 that has an E2 immediately after it, (v) total duration of E1, and (vi) mean duration of E1, no significant differences (*P* values in Table 2) were observed among all 4 treatments. For phloem ingestion analysis, data from the following variables in Table 2 were compared: (vii) number of E2, (viii) number of sustained E2, (ix) total duration of E2, (x) mean duration of E2, (xi) duration of longest E2, and (xii) percentage of E2 longer than 10 min. Sustained E2 ingestion in whiteflies can occur for a long period (Zhou 2014); therefore, we adopted the following procedure in the analysis of all these E2 related variables: if a recording was artificially terminated at E2 phase, the data were adjusted to include the annotation taken beyond the 4 h mark till the end of the ingestion period, or up to the 6th hour of the feeding event, depending on whichever occurrence came first. For artificial termination of probing behaviors such as pw or nonprobing phases, we did not make any adjustments as these events were shorter and would not affect the analysis. In all 6 variables related to phloem ingestion, no significant differences were observed among all 4 treatments (*P* values in Table 2), suggesting a similar level of phloem acceptability of colony-hosts and new-hosts by whiteflies. As one would reasonably expect, both duration of the first phloem phase (*P* = 0.41) and total duration of phloem phase (*P* = 0.71) were also not significantly different among the 4 treatments (Table 2, variables 13 and 14) consistent with the conclusion made above.

## Discussion

Whitefly feeding, a highly complex behavioral process associated with many variations of waveforms in EPG recordings, is associated with the activities of the stylet bundle as it transitions from outside the plant into the multicellular layers within in search of nutrient-rich phloem sap. No matter the complexity of the feeding behaviors and arena, it remains clear that to date, the feeding behaviors of only a few whitefly species, including those in the *B. tabaci* species complex, have been investigated by EPG (Janssen et al. 1989, Lei et al. 1998, Jiang et al. 1999, Jiang and Walker 2001, Jiao et al. 2014, Zhou et al. 2017, Milenovic et al. 2019). For those that have been studied, important aspects related to probing adaptation remain unexamined. Against the backdrop of these complexities and gaps in information, it was, therefore, important to investigate the feeding behaviors of the NW whitefly with a high level of granularity. To this end, we examined an extensive number (over 41 in total) of EPG variables, both individually and as a composite of multiple related variables, in order to gain an all-encompassing view of its feeding strategies under different plant environments.

The Napus and Rapa whiteflies used in this study have been maintained on their respective colony-hosts for many generations. They can produce eggs that progress through all 4 stages of nymphal development and, eventually, emerge as adults. Thus, they are ideal for addressing the questions raised in this study. The data for variables relevant to early probing behaviors as well as the probing and nonprobing patterns during pw phases (Figs. 2 and 3), indicated that whiteflies exhibited a unique probing profile on the plant (*B. napus* or *B. rapa*) used for EPG recording, irrespective of the whitefly (Napus or Rapa) tested. Moreover, whiteflies on a new-host (Rn and Nr) quickly adjusted and took on the probing behaviors of whiteflies reared on that colony-host (Nn and Rr, respectively), indicating that whiteflies could discern and adapt their probing behaviors to a previously unexperienced plant environment. Furthermore, a composite of variables with analyses presented in the form of a heat map (Fig. 3), showing a visual contrast of specific probing and pw phase behaviors produced on the 2 plants regardless of which whitefly (Napus or Rapa) the plants are presented with, further provides evidence that the plant on which *B. tabaci* NW feeds can trigger a set of unique and predictable probing patterns. Understandably, the probing behaviors seen on these plants are the result of the mechanical movement of the stylet bundle and secretion of salivary components (which come into an intimate interaction with plant components) en route to the phloem (Miles 1972, Xu et al. 2019, Walker 2022). The movement of the stylet bundle may be guided by either positive or negative stimuli from the plant (Lei et al. 1998). Evidence of the latter comes from studies showing that plant resistance factors present during pw phases can deter the whitefly from finding the phloem (Jiang et al. 2001). In this regard, the contrasting probing and pw phase behaviors between *B. rapa-* and *B. napus-*fed whiteflies would also suggest that whiteflies encountered more deterrence on the former than the latter.

In any given allocated EPG recording time, longer durations spent in nonprobing or pw phases, which include xylem phases, are possible signs of a less acceptable host as these measurements suggest that the whitefly experiences difficulties in locating the phloem (and thus has less time feeding in the phloem). Given this context, further analysis of data generated by whiteflies feeding on the colony- and new-host plants was performed using a composite of variables shown in, or deduced from, Fig. 4—i.e., total duration of probing, total duration of pw phase, total duration of nonprobing, total duration of nonphloem phase (= total duration of pw phase + nonprobing; for whiteflies that reached phloem phase), total duration of phloem (deduced from total duration of probing—total duration of pw phase),—that provide insights into 3 major activities, i.e., nonprobing, pw phases, and phloem phase, on which a whitefly expends most of its energy during an allocated feeding period. The data generated by whiteflies on their respective colonized hosts (Nn and Rr), showing that there was no significant difference in these variables, suggested that both whiteflies exhibited comparable host acceptance. On the new-hosts, data generated by whiteflies (Rn and Nr) for these variables were also not significantly different, with 2 exceptions—the total duration of probing and total duration of nonprobing (Fig. 4). A significantly longer total nonprobing duration and a shorter total probing duration were observed for Nr, whereas the opposite was observed for Rn. These results suggest that the *B. rapa* plant’s deterrence against a whitefly that has not adapted to it is significant. This conclusion is also supported by the early probes data (Fig. 2C), showing that more time was spent on the second nonprobe for Nr than Rn.

Accessibility to a constant supply of nutrient-rich diet is key to ensuring the survival, and hence, species continuity, of all living organisms. For hemipteran insects like whiteflies, this means being able to use their stylets to tap into and ingest sap from the phloem of a suitable plant host. In EPG parlance, a longer duration in achieving the first pd and phloem phase indicates more challenging pw phases and deterrence against the phloem-seeking activities of the whitefly. Given this context, in-depth analysis of data generated by whiteflies feeding on colony- and new-host plants was performed using 10 variables that examined the duration whiteflies took to achieve phloem phase and phloem ingestion (Table 1). The data generated by whiteflies on their respective colony-hosts (Nn and Rr) or new-hosts (Rn and Nr), showing that there was no significant difference in these variables, suggested that the ability to achieve phloem phase was comparable between both Napus and Rapa whiteflies. Within the phloem, whiteflies salivate before ingestion; in EPG parlance, a longer salivation duration and a shorter phloem ingestion duration are signs of poor phloem acceptability. The data generated by Napus and Rapa whiteflies on their respective colony-hosts (Nn and Rr) or new-hosts (Rn and Nr), showing that there was no significant difference in the phloem phase-related variables (Table 2), suggested that phloem acceptance was comparable between both whiteflies.

The ability of Napus and Rapa whiteflies to achieve phloem entry and sustained ingestion equally well on their colony-hosts and the new-hosts despite facing some deterrence in the pw phase in *B. rapa* plants is reminiscent of the behaviors of whiteflies feeding on tomato plants expressing the *Mi1.2* gene. Mil.2 is involved in probing deterrence; while whiteflies feeding on *Mi1.2* gene-expressing plants are challenged in locating the phloem, sap ingestion proceeds unhindered once they reach the phloem phase (Jiang et al. 2001). Intriguingly, colonies of NW whiteflies reared on *B. rapa* plants always appear to be more robust (populated) than the those reared on *B. napus* plants (our personal observation). Based on our results, the difference in colony vigor is unlikely due to a difference in the whiteflies’ ability to probe or acquire phloem sap in their respective colonized plants, but rather a difference in other factors such as the phloem sap content.

The findings from this study are an important reminder for caution in performing EPG studies on a plant species using whiteflies that are reared on another (different) plant species. Using whiteflies reared on 1 plant species to perform EPG on a different plant species is commonly seen in EPG studies. This is understandable given that it can take a very long time to establish a colony. To compensate for the time taken by a whitefly to adjust to a unfamiliar, new-host plant, exploratory EPG recordings are carried out to determine the optimal recording duration, and longer recording times are often allocated (Lei et al. 1997, Van Helden and Tjallingii 2000). Another strategy is to use whiteflies reared on a plant species that is completely different from all the plants used in the EPG experiments so that the acclimation time is equally applied to all treatments. In our study, given that EPG was performed on a colony-host of the whitefly, a 4-h recording was allocated. Whiteflies can reach sustained phloem phase from as low as 16 min to over an hour from the beginning of a recording (Lei et al. 1997, 1998, 1999, Jiang et al. 1999, Stafford et al. 2012). In our experiments, both Napus and Rapa whiteflies quickly recognized their respective colony-hosts and each immediately fed in a distinct pattern. Allocating a 4-h recording time on the new-hosts was also logical, for this allowed us to assess a whitefly’s ability to adapt to a plant environment different from that of its colony-host. Given that NW whitefly is deemed a less aggressive member of the *B. tabaci* species complex, it is remarkable that it is so highly adaptable on an unfamiliar, new-host. It is possible that the adaptation was not a huge challenge in this case because both Napus and Rapa whiteflies were reared on *Brassica spp*. (*B. napus* and *B. rapa*, respectively). However, the significantly longer duration of nonprobing and shorter duration of total probing of Napus whiteflies on *B. rapa* (Nr) compared to that of Rapa whiteflies on *B. napus* (Rn) (Fig. 4) suggest that rearing Rapa whiteflies on the former (*B. rapa*) (Rr) can improve their acceptance of this host as far as these 2 variables are concerned (Fig. 4).

## Supplementary Material

ieae084_suppl_Supplementary_Figures_S1

ieae084_suppl_Supplementary_Figure_Legend
