# Supplementary material for: Adaptation of feeding behaviors on two Brassica species by colonizing and noncolonizing Bemisia tabaci (Hemiptera: Aleyrodidae) NW whiteflies
Source: J Insect Sci. 2024 Sep 3;24(4):20. doi: 10.1093/jisesa/ieae084 (PMC11369500; doi:10.1093/jisesa/ieae084)
Supplement: ieae084_suppl_Supplementary_Figures_S1 [file ieae084_suppl_supplementary_figures_s1.pdf]

Supp. Fig. S1

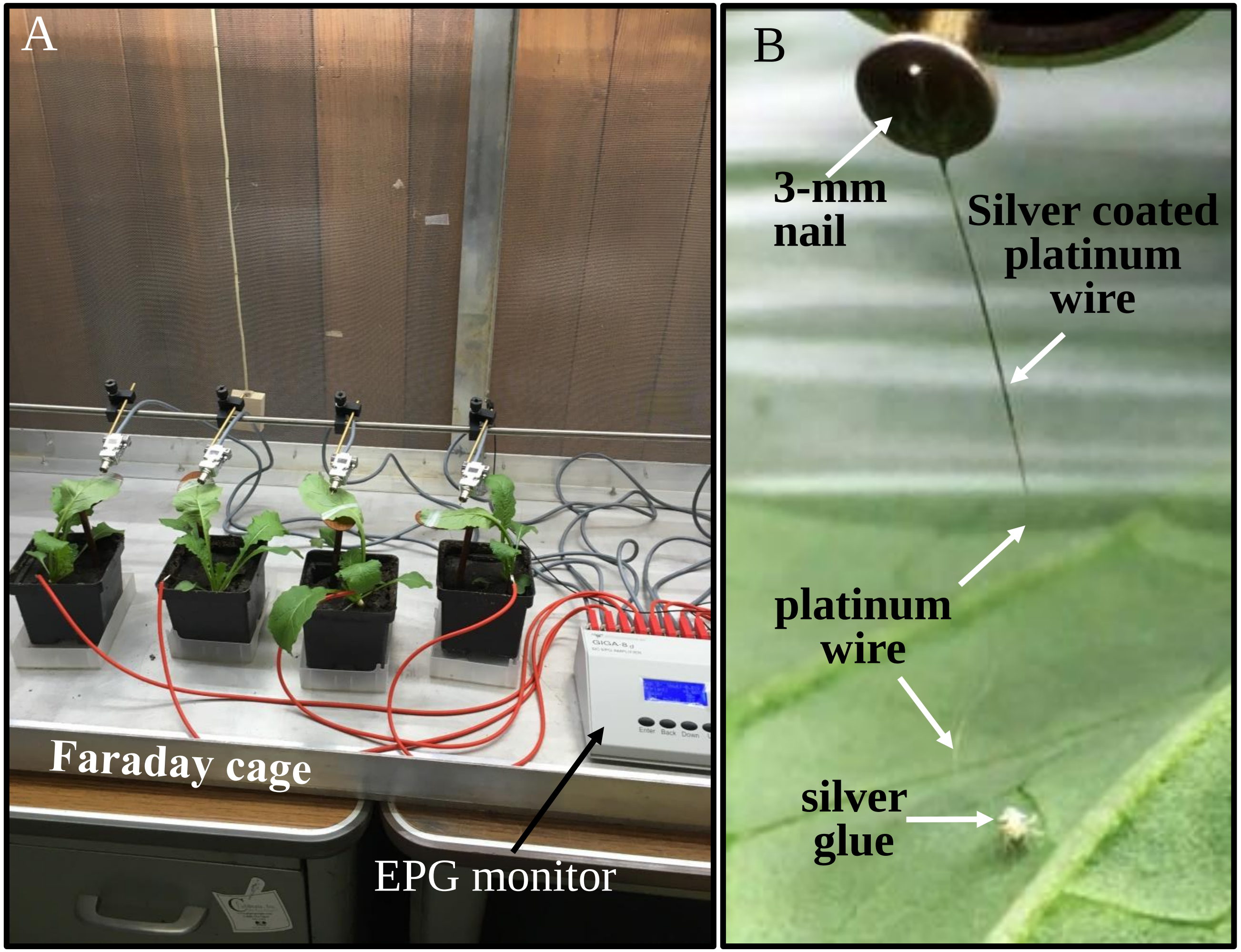

Bemisia tabaci NW whiteflies in an EPG recording set up. (A) Picture of EPG recording in progress using the Giga-8D DC-EPG monitor with eight channels (four shown in picture) being used simultaneously. Potted plants used for the EPG recording were placed inside a Faraday cage (door lifted and not shown) with the EPG monitor (partial view). A voltage was applied to the plant through the output wire (red) inserted into the soil of each pot. A platinum-wired NW whitefly was placed on the abaxial side of a leaf of the individual plants. (B) Close up of a platinum-wired NW whitefly on the abaxial side of the leaf.
